# A concept in flux: questioning accountability in the context of global health cooperation

**DOI:** 10.1186/s12992-014-0073-9

**Published:** 2014-12-09

**Authors:** Carlos Bruen, Ruairí Brugha, Angela Kageni, Francis Wafula

**Affiliations:** Royal College of Surgeons in Ireland, 123 St Stephens Green, Dublin 2, Ireland; Centre of Research Excellence in Rural and Remote Primary Health Care, Bendigo, Australia; Centre for Remote Health, Flinders University and Charles Darwin University, Alice Springs, Australia

**Keywords:** Accountability, Global health, Health policy analysis, Global Fund to Fight HIV/AIDS, Tuberculosis and Malaria

## Abstract

**Background:**

Accountability in global health is a commonly invoked though less commonly questioned concept. Critically reflecting on the concept and how it is put into practice, this paper focuses on the who, what, how, and where of accountability, mapping its defining features and considering them with respect to real-world circumstances. Changing dynamics in global health cooperation - such as the emergence of new health public-private partnerships and the formal inclusion of non-state actors in policy making processes - provides the backdrop to this discussion.

**Discussion:**

Accountability is frequently reduced to one set of actors holding another to account. Changes in the global health landscape and in relations between actors have however made the practice of accountability more complex and contested. Currently undergoing a reframing process, participation and transparency have become core elements of a new accountability agenda alongside evaluation and redress or enforcement mechanisms. However, while accountability is about holding actors responsible for their actions, the mechanisms through which this might be done vary substantially and are far from politically neutral.

Accountability in global health cooperation involves multipolar relationships between a large number of stakeholders with varying degrees of power and influence, where not all interests are realised in that relationship. Moreover, accountability differs across finance, programme and governance subfields, where each has its own set of policy processes, institutional structures, accountability relations and power asymmetries to contend with. With reference to the Global Fund to Fight HIV/AIDS, Tuberculosis and Malaria, this paper contributes to discussions on accountability by mapping out key elements of the concept and how it is put into practice, where different types of accountability battle for recognition and legitimacy.

**Summary:**

In mapping some defining features, accountability in global health cooperation is shown to be a complex problem not necessarily reducible to one set of actors holding another to account. Clear tensions are observed between multi-stakeholder participatory models and more traditional vertical models that prioritise accountability upwards to donors, both of which are embodied in initiatives like the Global Fund. For multi-constituency organisations, this poses challenges not only for future financing but also for future legitimacy.

## Background

Writing in his weekly column, The Lancet editor Richard Horton remarked that global health has experienced a recent revolution in accountability, with improved metrics increasingly linked to politics and decision-making in ways that seek to create “the right political conditions for data to have an impact on health and health policy” [1]. Substantial changes in the global health landscape since the late 1990s have served to intensify demands for greater accountability, including: increases in funding for specific diseases or interventions; a growth in the number of state and non-state actors involved in the making, financing and implementation of health policies; and more diverse channels for funding global health projects and programmes [2]. However, as both a concept and in practice, accountability is infused with different meanings, criteria and standards depending on who uses the term, whether managers, policy makers, researchers, advocates, or health professionals. Consequently, it has become a malleable, contested and widely extended concept, in so far as it is applied in different ways across a multi-layered and non-linear global health system within which diverse lines of accountability between different sets of actors compete or complement one another.

Accountability in the context of these converging global health trends sets the background for this discussion paper. We ask what is meant by accountability (or at least what are the different understandings), who is to be held accountable, and how, both conceptually and with reference to real-world circumstances. Accountability in practice is often underpinned by a Principal-Agent logic: based on lead-subordinate roles, relations are structured to incentivize Agents to act in the interests of the Principal or lead [3]. This is an expression of an institutional power relation, whereby the Principal confines the range of choices available to the Agent, thereby directing the actions of the Agent to fulfil the priorities of the Principal. Accountability mechanisms, such as monitoring and evaluation activities, can play a functional role in these relationships, in that they may be put in place to mitigate unwanted opportunistic behaviour of Agents. Authority is assigned to the Principal actor to seek rectification or enforce sanctions when goals are not accomplished or responsibilities not met [4,5]. More sophisticated forms of Principal-Agent models set precise targets in projects, often with a threat of penalties or punishment for poor performance, in order to reduce the risk of capture and improper use of funds in foreign aid relations [6], or to enhance health system performance and service delivery [7].

Viewed critically, however, such a narrow application of accountability corresponds closely with what Fidler describes as ‘foreign policy preferences that ruthlessly winnow complex problems into defined tasks with measurable targets’ [8]. Moreover, compliance and punishment often dominate over other possible responses, most notably opportunities for information generation, learning and organisational change. While remarkably pervasive, the empirical reality of global health does not always lend itself to readily identifiable Principals and Agents, nor does this model adequately reflect the multiple accountabilities that actors contend with [9,10]. Accountability in global health is complex and subject to regular re-interpretation. Should, for example, the World Health Organization or the World Bank be accountable to the states who created and continue to fund them, or should these organisations be accountable to the people most affected by their policies? How can multiple stakeholders in these and other organisations hold one another to account? Where does the locus of responsibility lie? And who constitutes the legitimate source of authority in a particular situation for judging if responsibilities have been met?

In the section that follows, we question the *who*, *what, how*, as well as the *where* of accountability. We examine the changing and distributed nature of global health cooperation, and consider this with respect to some of the primary constituencies involved, e.g. populations affected by diseases or programmes, governments, multilateral organisations, philanthropies, private or non-governmental organisations (NGOs); and in particular relatively recent public-private partnerships like the Global Fund to Fight AIDS, Tuberculosis and Malaria (hereafter Global Fund) that bring many of these actors together. Our aim here is to reflect on the concept and practice of accountability in the context of global health cooperation, and to identify and chart some of its defining features. We then discuss these features in relation to one of the newer public-private partnerships to claim accountability as a core value, the Global Fund.

## Discussion

### Changing dynamics of global health cooperation

A commonly accepted definition of accountability is the condition of being responsible and answerable to someone for meeting performance or other activities, measured against a set of standards [7]. This model is based on relations between at least two different actors with different levels of resources. These relations assume the existence of a higher authority with an oversight function [11]. By extension, this implies ‘that some actors have the right to hold other actors to a set of standards, to judge whether they have fulfilled their responsibilities in light of these standards, and to impose sanctions if they determine that these responsibilities have not been met’ [12]. In international relations, some consider individuals as the ultimate source of responsibility - others, however, argue that collective entities like states or organisations also qualify as responsible agents who have interests, deliberate on courses of action and their consequences, have specific aims and duties, and should thus be accountable for their actions [13].

Under principles of international law, the state has primary responsibility for ensuring the health needs of its population is met, including responsibilities for ensuring adequate health sector funding, for governing well, and for addressing the socioeconomic determinants of health [14]. Additionally, extraterritorial duties oblige wealthier countries to support those countries unable to ensure the basic health needs of their populations. While this can take the form of financial or other assistance, issues of jurisdiction, division of responsibility or the regulation of non-state actors, among other things, can make this a highly contested and difficult issue in national, bilateral and multilateral arrangements [14-17].

While states are deemed responsible for health within their borders, they have delegated some authority to inter-state organisations like the World Health Organization (WHO) to address health issues from a global level. The WHO, for example, is required to demonstrate internal accountability within its secretariat and operational structures, and is, more broadly, formally accountable to member governments, who in turn can influence strategic policy decisions using the 'one state, one vote' system at the World Health Assembly. In practice, representative decision-making in multilateral organisations can be compromised by organisational structures that allow for key agenda setting decisions to be made outside of the representative structures or through the provision of multiple ear-marked funds that distort broader sectorial strategies [18].

As a result, critics have long argued that power differentials make multilateral organisations like the WHO or the World Bank more accountable and responsive to the interests of some members over others, where small groups of powerful states exert influence through formal and informal channels [10,18,19]. Furthermore, while the WHO may claim to be responsive solely to member governments, the changing dynamics of global health means that non-state actors like the Bill and Melinda Gates Foundation, large companies or NGO networks have a substantial influence over the financing, decision-making or implementation activities of inter-state organisations like the WHO. The WHO itself continues to struggle politically and organisationally with this issue, encountering difficulties in formally codifying its engagement with non-state actors. In the eyes of some, it risks becoming irrelevant and outpaced by younger rivals like the GAVI Alliance and the Global Fund who have formally incorporated non-state actors in their decision-making structures [20].

The challenges faced by the WHO reflect fundamental changes in the dynamics of global health cooperation since the 1990s, changes that impact a wide range of actors and have in turn had knock-on consequences for and initiated reinterpretations of accountability. NGOs, philanthropic organisations and for-profit companies have seen their power to influence extend across state borders, into traditional inter-state structures, as well as becoming incorporated into newer global health initiatives (GHIs) [21]. This has been coupled with demands for better accountability of these actors, along with the development of newer accountability methods, such as peer regulation initiatives, voluntary codes of practice or community accountability and transparency initiatives [22-25]. The very nature of global health cooperation has changed, shifting from vertical tiers of state representation characterised by the WHO and the UN system more broadly, towards more horizontal models of participation of selected stakeholders in public-private partnerships [26]. GHIs like the Global Fund and the GAVI Alliance exemplify these new forms of global cooperation that bring together state and non-state actors, formalising engagement for the purposes of resolving specific health policy problems such as financing the control of infectious diseases. While often praised for being more participative and transparent relative to other global organisations, questions remain regarding to whom global public-private organisations are in fact accountable and responsive [27-29].

The rise of non-state actors and GHIs has revolutionised the global health landscape. However, their influence and incorporation in global health decision-making structures has raised concerns about overlapping and competing mandates between public and private actors, as well as risks involved with diluting responsibility across a widening set of actors [26,30,31]. This is not to suggest that accountability structures of the past were somehow stronger, or that public actors like the WHO or states are implicitly more accountable or legitimate. Rather, changing global health dynamics and actor relations not only challenges traditional accountability structures but has also brought about a reframing process, one that needs to be carefully observed. This is necessary to avoid disempowering the concept, such as reducing it to ideas of transparency or technocratic monitoring, or allowing it be strategically employed as an instrument to legitimize particular issue areas or the activities of specific actors [32].

### Changing dynamics of accountability

Accountability is a powerful concept. It is normative, in so far as it is indicative of desired conditions, such as due process, transparency or participatory decision-making that will lead to better health or other outcomes [9]. Accountability mechanisms and processes are in turn the means through which these desired conditions might be realised. Accountability processes and mechanisms are not politically neutral however – they may reinforce existing power relations or they may be agents of change. Rather than simply assuming therefore that more monitoring, regulation or sanctions will address problems of accountability, ‘one must look to the effects of those mechanisms to understand their impacts and operations, rather than the rhetoric that motivates and accompanies them’ [9].

In the context of global health cooperation, accountability is not simply a binary relationship where one set of actors is held accountable to another. Real-world accountability involves a multipolar relationship between a large number of stakeholders with varying degrees of power and influence, where not all interests or preferences are realised in that accountability relationship [32]. Spatial metaphors are sometimes used to suggest competing lines of accountability between different groups of actors. Vertical lines of accountability flow ‘upward’, such as to funders or state agencies, or ‘downward’, to citizens or those affected by services delivered, raising related questions as to whether or not enforcement should rely on top-down or bottom-up mechanisms [9,33]. Horizontal lines refer to inter-institutional mechanisms, such as between executive and legislative state agencies, or between actors in a delegate body or board [34,35]. Hybrid forms of accountability attempt to bridge the vertical-horizontal divide. Also termed diagonal accountability, this can refer to direct citizen engagement with more powerful public or private institutions through mechanisms like joint state-civil society monitoring initiatives or citizen auditing [34].

A single actor may at any one time be expected to give account to numerous other actors, such as NGOs to their donors, to their board, to the people affected by their actions, or to the legal system in the country they are registered or country they operate in. NGOs, like other actors, are often caught in a dilemma between meeting the accountability demands of one set of actors, such as donors, while also being expected to give account to communities they engage [33,36]. It is in this sense that accountability is a multipolar relationship, one made more complex by the fact that on occasion actors can be both objects and agents of accountability, required to be answerable while also seeking to hold others to account [37].

Based on literature covering financial control, public sector management reform, and governance, Brinkerhoff specifies three types of accountability required for holding health actors to account [7]. Financial accountability, the most commonly understood type, “concerns tracking and reporting on allocation, disbursement and utilization of financial resources, using the tools of auditing, budgeting and accounting‘’ [7]. Equally importantly, however, is that actors must also be accountable for performance, which includes demonstrating results against agreed programme targets. Finally, Brinkerhoff outlines the importance of political accountability, and equates it to governments delivering on electoral promises and responding to the needs of citizens, with state-based political processes and elections referenced as the main accountability channels.

While this three-way classification of accountability types is systematic, it does not take into account wider health policy processes influencing accountability dynamics. In particular, the national and international actors who make or influence policy are largely neglected here, as are the asymmetric relations between actors that may lead to modes of accountability that are skewed to favour the interests of more powerful actors. It is necessary to also ask questions regarding who gets to decide on or design accountability interventions, to set the benchmarks or targets against which interventions or decisions should be evaluated, and whether or not efforts to improve accountability actually achieve their purported aims. This means recognising ‘the relationships, demands and power plays among actors…[where] numerous types of accountability battle for recognition and legitimacy’ [9].

### Mapping accountability mechanisms and policy subfields

The provision of information to people trying to hold power-wielders to account, coupled with the ability to demand answers and impose sanctions, appear in the global governance and international relations literature as core components of accountability and as mechanisms for guarding against abuses of power [12,35]. Within this literature, Hesselmann for instance considers transparency, answerability and enforcement as defining the first and second stages of accountability, where each stage demands progressively more from the actors being called to account [32]. Accountability is only achieved when an accountability-giver adapts its behaviour in response to enforced sanctions or rewards, which is the third stage of accountability. Weisband and Ebrahim similarly identify four elements of accountability, namely transparency, answerability, compliance and enforcement, each building on the other and requiring enforceability if accountability is to be realised [9].

Added to this, Woods and Narlikar note that mechanisms for monitoring and evaluation are a prerequisite, alongside some degree of transparency, and the ability to enforce rules and seek redress [35]. Monitoring and evaluation provides a means to generate information on processes, decisions or outcomes of an institution or programme, while transparency mechanisms facilitate the availability of that information. It also provides a critical link between accountability, organisational learning and behaviour change. This has its challenges, however, including the need for appropriate mechanisms to be in place that ensure information generated from evaluations finds its way back into decision-making processes, and to ensure that the evaluation processes themselves are not simply measuring performance to enforce compliance or to punish, but are also a means for improvement [38].

A new accountability agenda has emerged in response to democratic failures and pressures for generating new ways of holding powerful actors to account, most notably in the form of increased participation in formal institutions of decision-making and oversight [37]. At the global level, concerns of a ‘democratic deficit’ have led to participation becoming a core element of the new accountability agenda. As part of this new agenda, accountability is being reframed to include participation as a means to make power-wielders answerable to those affected by their actions, or holding them to externally verifiable standards or benchmarks. Moreover, the legitimacy of international organisations is increasingly viewed as dependent on the participation of actors beyond those who have formal authority over an organisation; and it is presented as a way to make these organisations more effective, develop better informed policy and improve implementation [12,19,35].

Burall and Neligan outline four broad elements required to improve conditions for effective information provision and enforceability, identified above as basic requirements for accountability [10]. Termed both ‘dimensions’ and ‘principles’ by the authors, these elements encompass broad mechanisms, that is, mechanisms for participation, for transparency, for monitoring and evaluation, along with complaints and redress procedures for ensuring compliance. For participation to be effective, structures need to be in place that provide relevant stakeholders with opportunities to engage in decision-making, setting standards and rules, monitoring processes or responding to problems arising in finance, programme or governance processes. Transparency refers to recording, reporting and publishing of information in a timely manner, including either proactive or reactive disclosure of information. Monitoring and evaluation of organisations and policies is important for assessing the operations and effectiveness of programmes or organisations. Complaints and redress mechanisms support compliance and enforcement activities, such as judicial-style panels, ombudsman’s offices or citizen juries.

Burall and Neligan emphasise ‘the ongoing, participative nature of accountability’ [10]. Increasing participation in the policy processes of international organisations is considered as offering transformative potential [9,35]. Participation in priority-setting, decision-making and oversight comes with its own challenges however, not least with regard to what form it should take, whether representative, delegated or direct; who should be involved, through what processes; as well as the feasibility of implementation at a global level [9,12,19]. New global health initiatives like the Global Fund are reflective of this changing demand, incorporating models of participation into their organisational structures that provide access for selected non-state actors in decision-making and over-sight at both global and country levels.

While the discussion above has concentrated on who, what and how, we treat the where of accountability not just in terms of specific institutional settings, but rather as action areas or sub-fields common to the broader global health policy field. As Buse and colleagues suggest, health policies provide the overarching frameworks for activities in global health, setting goals and shaping ‘courses of action (and inaction) that affect the set of institutions, organisations, services and funding arrangements’ [39]. Borrowing from social and policy theory [40,41], health policies can be viewed as both an outcome of and guiding framework for field activity. Considered this way, the global health policy field possess rules, functions and sets of issues deemed important to its occupants. It is occupied by actors who differ in terms of the amount and relative weight of economic, social, political and symbolic capital they possess. This structures the field by creating a hierarchy of relations that gives actors more or less influence over what health policy issues are deemed to be at stake, as well as determining who has the authority to hold others to account and how. And like any field, the global health policy field is comprised of interdependent subfields with a potential to effect change in one another. Three particular subfields of relevance to health policy processes are finance, programme and governance subfields, where each has a different focus and impacts on the others in a variety of ways. These action areas or subfields have their own subsets of policy-making issues to contend with, as well as their particular policy processes and accountability dynamics that constrain or enable different actors in different way. In other words, the finance, programme, and governance subfields each impact upon the overall ‘doing’ of global health policy and accountability.

As with the analysis of health policies [39], analysis of accountability in the three policy subfields must also examine and acknowledge the importance of actors, processes and contextual factors that shape and influence the implementation of accountability instruments. Beginning with the financial subfield, questions regarding the creation and implementation of financial accountability procedures, regimes and standards, and the values that are embedded within them, are as important as the procedures or regimes themselves. This might focus on, for instance: the design and selection of auditing and reporting tools to be utilised, who is authorised to use them and whose information needs are being served; or the processes and values underlying fiduciary principles that define what and who can be financed, and from what source. Secondly, the programme subfield refers to more than monitoring and evaluating programme outcomes. Also of relevance are the processes leading to the incorporation of particular issues into programmes in the first instance; the frameworks and specific targets for evaluating programmes tasked with addressing those issues; along with the policies and administrative structures that shape the supply and delivery of services and the ability of actors to meet programme targets. Instruments of accountability in programme activities are not politically neutral, and the design and effects of those mechanisms must also be examined. In the case of programme monitoring and evaluation, for example, these instruments may in fact favour the information needs of some actors, such as donors, possibly to the detriment of and financial cost to governments in receipt of funds, as has sometimes occurred in HIV/AIDS programming [42].

Finally, issues of governance are fundamental to the health policy process, where political accountability involves much more than states being accountable to citizens. Once the reserve of governments, there has been a rearrangement of responsibilities and inclusion of non-state actors in collective health decision-making settings, a change that has been summed up “as representing the transition from a world of governments to a world of governance” [39]. Bureaucratic, supervisory, fiscal and legal mechanisms have been supplemented by newer market, peer and reputational instruments for holding power-wielders and those with less power to account for their actions [12]. This in itself is seen by some as a response to the ‘private turn’ in global governance and health, the rise of partnerships as legitimate governance actors, and the accountability challenges this has created [30].

Critics argue that governments and international institutions have compromised their own accountability mechanisms by entering into partnerships with private sector actors [16]. Others note however that partnerships have incorporated mechanisms to enhance their internal governance models in ways that may in fact be increasing the democratic nature of governance in global and country settings, such as through stakeholder inclusion or open-door board meetings [30]. These and other questions are of relevance to accountability in the governance subfield, the setting in which issues are prioritised, policies formulated, and where the governance infrastructure itself determines who is involved in these processes or how those involved can be held to account.

**Figure 1:**
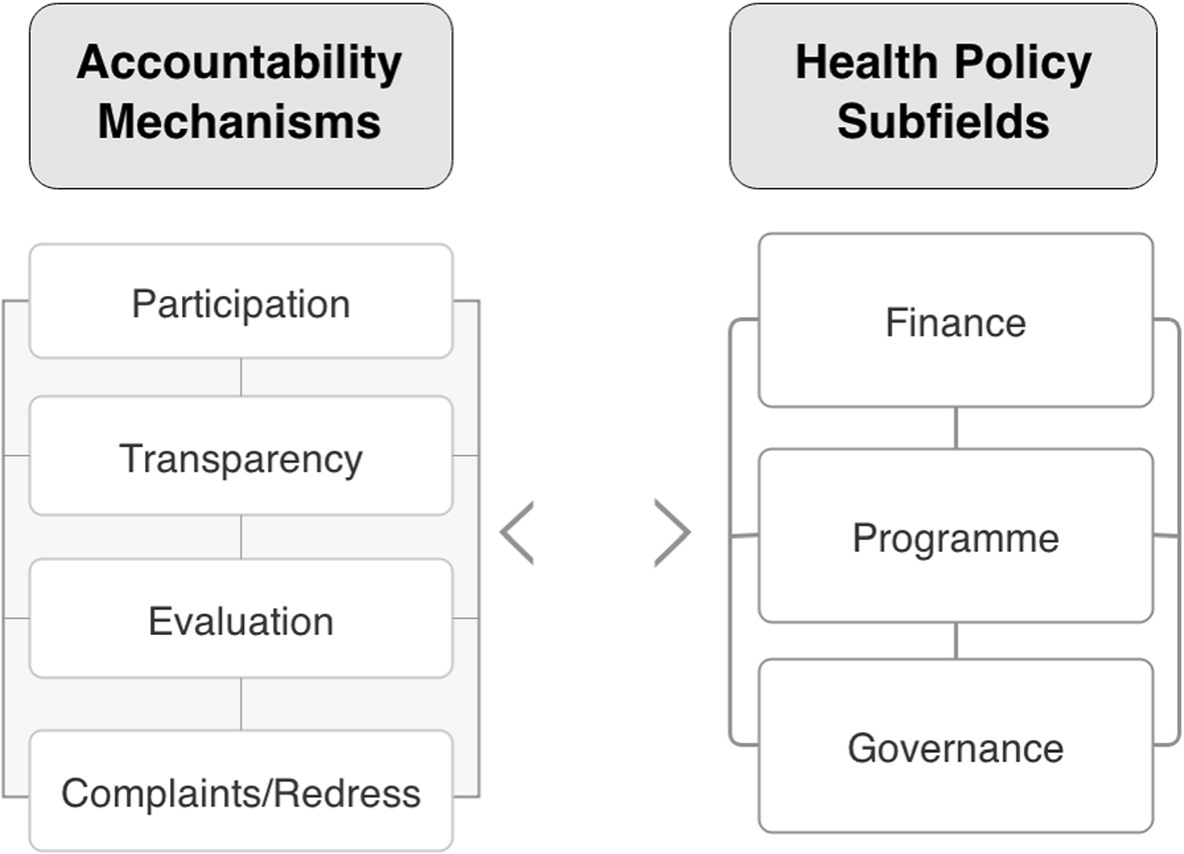
**Conceptual map of accountability.**

Brought together, the accountability mechanisms and health policy subfields identified in the discussion above provide basic elements for our conceptual map of accountability (Figure 1). We have approached accountability in the context of global health cooperation as a system of relations between different actors with varying degrees of power and influence. Accountability is frequently complex and not always reducible to linear relationships that are formed by the understandable need for making recipients accountable for the use of donor funds. As the following section illustrates, accountability relations between actors are complex, dense, and contingent on their context. In practice, accountability has been characterised as a ‘political theatre’ where institutional structures, power asymmetries and inter-organisational dynamics combine to ensure that “no single form of accountability dominates” [9].

### Illustrating accountability relations: the Global Fundcase study

The Global Fund provides a useful entry point to illustrate multiple accountability relations, while the subfields identified (finance, programme, and governance) provide an organising frame to discuss accountability issues, and to highlight how accountability is an ongoing process of learning, change and contestation. The Global Fund is representative of the changing dynamics in global health cooperation described earlier, and has incorporated elements of the new accountability agenda into its operations, such as extending participation beyond traditional state actors at the highest levels. The Global Fund Board, for example, includes representatives from donor and recipient governments, NGOs, affected communities, multilateral organisations, the private sector, and philanthropic organisations. As the supreme governing body, the Board develops organisational strategies, oversees governance and finance activities, as well as assesses performance and risk. In comparison to other global health organisations, the Global Fund has been described as one of the most open and transparent [43,44], rated sixth in the 2013 Aid Transparency Index of donors [45]. The organisation has been credited with contributing to stronger health governance, increased participation of diverse actors in health policy processes, and improved responsiveness to country needs [46].

The purpose of the Global Fund is to attract, manage and disburse additional resources to countries, and does not implement or manage programmes itself. To that end, the Global Fund relies on governments, NGOs and other actors in countries to develop proposals and implement programmes. From its inception, the Global Fund’s approach to country engagement has been different to most other international health organisations, opting for country partnership mechanisms rather than having country offices. Country Coordinating Mechanisms (CCMs) are the preferred mechanism for proposal development and entering into contractual relationships with programme implementers. The CCM is the central governance mechanism at country level, and is envisioned as a country-level mirror of the public-private Global Fund Board. The CCM is primarily responsible for coordinating the development of proposals and submitting them to the Global Fund, as well as being responsible for submitting funding requests after each evaluation period. Since the launch of the Global Fund strategy for 2012–2016, the CCM has been charged with an increasing grant oversight role, including overseeing grant implementation by the CCM-nominated Principal Recipient (PR), and verifying that reporting and other requirements are met [47].

While the CCM represents the governance structure in countries, the PRs and Sub-Recipients (SRs) represent the implementing functions. The Local Fund Agent (LFA) represents an extension of the assurance function, verifying financial and programmatic reports submitted by the PRs to the Global Fund. LFAs are selected through competitive bidding, and generally include global auditing companies such as KPMG and PricewaterhouseCoopers. PRs are legally responsible to the Global Fund, and must cooperate with LFAs and CCMs. PRs report to the Global Fund Secretariat via the LFA and the CCM, while LFAs report directly to the Global Fund Secretariat. From time to time, the OIG may review activities in countries for the purposes of identifying misuse of funds and detection of fraud, waste or mismanagement in grants.

### Accountability framed

Before covering the three subfields, we first outline how accountability is framed by the Global Fund, and the spaces where formal authority lies. The Global Fund Framework Document interprets accountability in a specific way, as requiring “sound processes for specifying, tracking and measuring programme results to ensure a sufficient level of accountability” [48]. Crucially, it notes that the “future financial viability of the Global Fund will depend on being able to demonstrate results, initially in terms of coverage of activities and subsequently in terms of outcomes”, whereby a system of accountability is “needed to provide incentives to grant recipients to achieve more, faster, and better results”. Grantees are thus charged with delivering results, and need to be “accountable to government, private sector and foundation donors (for the use of funds, achievements of results)”.

The 2013 Global Fund Annual Financial Report details the commitments of donors during the third Replenishment Round, 2011–13. Commitments from donor governments represented 93% of overall contributions, the largest of which was from the United States Government. Foundations and the private sector committed 5% of overall contributions, while the remaining 2% came from the Affordable Medicines Facility - Malaria (AMFm), an initiative funded by the UK and Canadian governments, UNITAID and the Bill & Melinda Gates Foundation. The future viability of the Global Fund therefore is dependent on incentivising grantees to generate results that satisfy the expectations of its donors, and donor governments in particular who represent by far the largest contributors to the organisation.

Not surprisingly, this positions donors as extremely powerful actors in the accountability relationship. It is reflective of a two-stage upward movement of financial accountability, firstly from grantees to the Global Fund, where enforcement relies on top-down monitoring to identify financial mismanagement or corruption, discussed later. The second stage is onward accountability to donors, where withholding or withdrawing financial support provides a powerful sanctioning and redress tool and can be employed to change the behaviour of the organisation to meet the demands of its donors. Barnes and Browne see in this model of accountability an inbuilt tension and potential imbalance of political influence: the Global Fund, they argue, shares with traditional business models a process of vertical prioritisation of accountability upwards to donors, rather than horizontally to the multisectoral partners involved [29].

**Figure 2:**
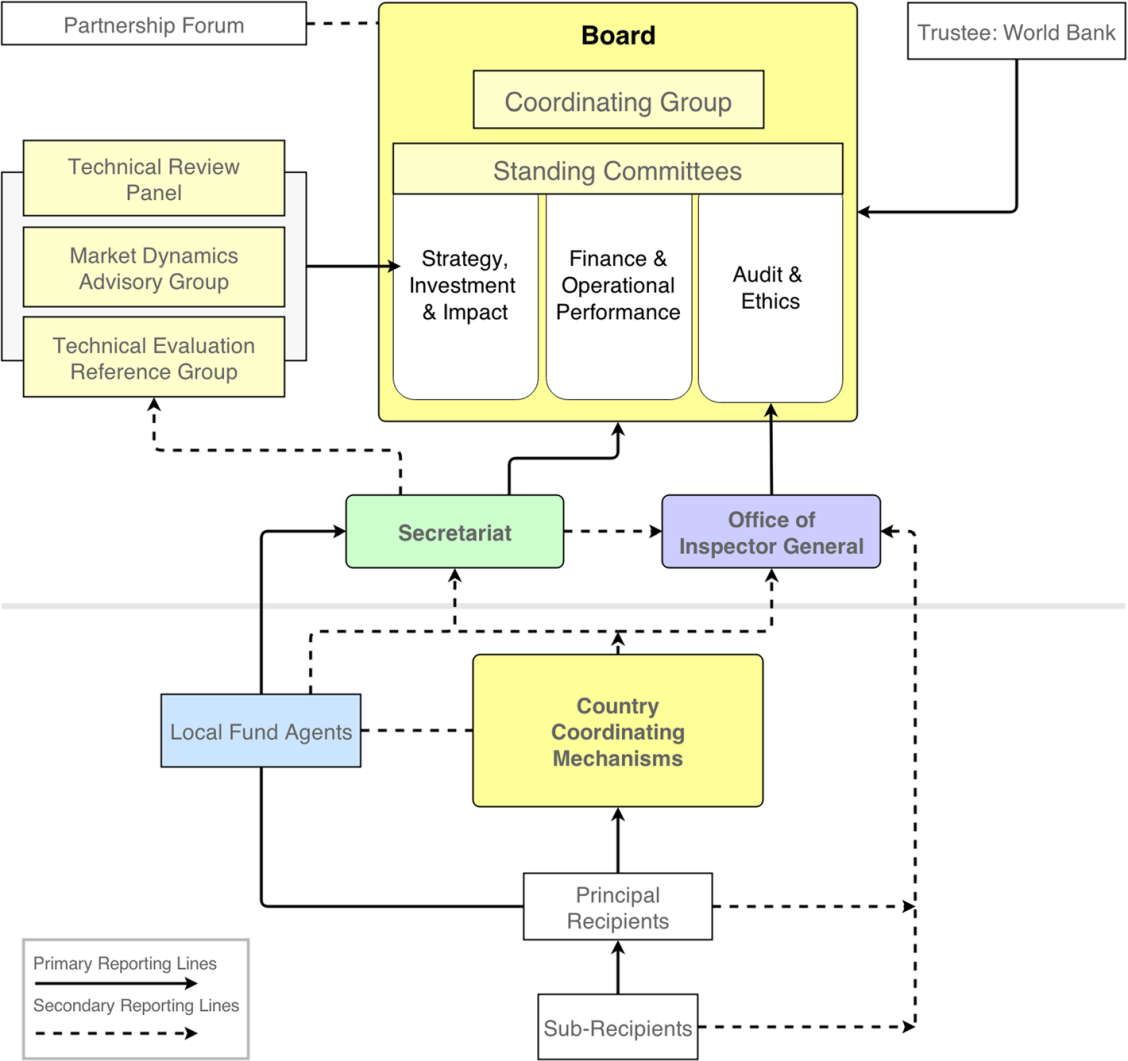
**Organisational and Reporting Structure of the Global Fund.**

Figure 2 highlights this vertical arrangement to illustrate who reports to whom, and who is vested with the authority to demand reports and information internal to the organisation. Primary reporting lines denote required and regular reporting (solid lines), and are usually contractually bound in grant or hiring agreements, or are built into the terms and conditions of engagement. Secondary reporting (dotted lines) is less regularised, and is usually in response to investigations or requests for information. Only some actors are empowered to request or demand information. The Secretariat, for instance, is not in a position to request reports on the operation of the Office of the Inspector General (OIG), while the OIG is only charged with reporting to the Audit & Ethics Committee of the Global Fund Board. The OIG on the other hand can investigate or audit the operations of any number of actors, including the Secretariat, and has the authority to access all books and records maintained by the Global Fund, and to seek any information from people involved in the organisation’s projects.

In some ways, the accountability structure of the Global Fund resembles a chain of technical Principal-Agent relations, from sub-recipients to donors. But while donors are certainly the most powerful set of actors, the ‘political theatre’ and inter-organisational dynamics across the finance, programme and governance subfields ensures this is more than a simple Principal-Agent system of control and compliance. We turn to these issues for the remainder of this section to describe some of the tensions and challenges between different actors in this relationship.

### The finance subfield

The Technical Review Panel (TRP), which is part of the global level governance of the Global Fund, plays an important role in the financial subfield, though its influence lies predominantly in the programme subfield discussed later. Established as an independent expert advisory panel, the TRP assesses requests for funding from countries, presents its recommendations to the Board for final decision, and thus starts a financial relationship between the organisation and those receiving funds. Once approved, grant agreements between the Global Fund and the PRs establish the terms and conditions for accountability in financial disbursement and use, procurement requirements, as well as financial and programme reporting. Financial and programme reporting are handled separately, though are linked at certain points along the process.

The World Bank, as Trustee, is responsible for managing Global Fund monies, including making payments to recipients at the instruction of the Secretariat, and reports on financial management to the Board. During its earliest years, all recipients of Global Fund finances had to provide regular financial reports through the CCM to the Trustee or an approved sub-trustee. The goal of the Fund during this period was heavily focused on a rapid expansion of resources, a rapid scale-up of programmes and quickly demonstrating results – reflected in the ‘raise it, spend it, prove it’ motto of the organisation’s first director, Richard Feachem. Within a few years however, concerns about financial accountability began to surface. By July 2005, the Global Fund Board agreed to establish an independent inspector office, with the Charter of the Office of the Inspector General approved at the 13^th^ Board meeting in April 2006.

The establishment of the OIG underscored the importance major donors to the Global Fund placed on upward financial accountability for use of their finances, and came about in part as a result of donor conditions placed on their financial contributions to the organisation. Prior to the Board agreement, the US Congress authorised withholding 25% of its contribution to the Global Fund until it took steps to implement conditions Congress deemed essential for improving the organisation’s accountability. The primary condition was the establishment of “a full time, professional, independent office which reports directly to the Global Fund Board regarding, among other things, the integrity of processes for consideration and approval of grant proposals, and the implementation, monitoring and evaluation of grants made by the Global Fund” [49]. Congress’ condition remained in place for some time after the establishment of the OIG, and it continued to enforce strong oversight of its implementation during the initial years.

The creation of the OIG was prompted by the sanctioning threat of the most powerful donor and reflected the power of this donor to force the Global Fund to adapt its behaviour and institutional composition. The existence of the Office, regardless of what triggered it, was also reflective of the Fund’s founding aim of ensuring grantees were held accountable for their use of donor finances, part of which rested on a commitment to transparency and availability of evaluation reports. However, apart from donor governments or organisations with the technical capacity to review these reports, few organisations or people in countries have been in a position to benefit from them, in part due to the complexity and quantity of data and reports that makes them less accessible or relevant for many [50]. Additionally, up until recently a clause in the OIG charter meant that only its external assurance and investigation work would be made public, with internal investigations (such reviews of the Secretariat) only available to the Executive Director and the Audit & Ethics Committee. Barring exceptional circumstances, the Global Fund Board decided at its 31^st^ Meeting in March 2014 that all external and internal reports of the OIG will be made public from now on, though past reports will not be released [51].

The OIG evaluation and auditing mechanisms are essentially funder-control mechanisms, regularly identifying what it characterised as “losses” and reporting these to the Global Fund and the Board. Once investigative reports are finalised, they are publically posted to the Global Fund website in pursuance of its transparency goal, and redress mechanisms are instigated to retrieve lost finances. The OIG has been criticized however for failing to put losses into the context of the overall country or Global Fund grant portfolio, creating an unbalanced picture that ultimately led to claims that the Global Fund itself was plagued with misappropriation and fraud [52]. Equally critically, the early years of the OIG was marred with its own controversies, including findings that it only partially conformed to international auditing standards, that there were substantive questions over the quality and professionalism of its work, and that there was no mechanisms in place for PRs to lodge a complaint against the OIG in cases where they felt they had been poorly treated [53,54].

Concerns raised by the OIG on losses to the Fund were picked up by the media in mid-2011. This triggered a suspension of funding from donors, the establishment of the High Level Independent Review Panel, and a series of substantial reforms following the Panel’s recommendations [55]. An evaluation conducted by the Panel confirmed that the system of fiduciary control developed in the founding years was inadequate and had not worked as well as intended. For example, it found that almost every grant had at least one financial management problem; that the weaknesses in PRs could have been identified earlier; and that significant governance, monitoring and evaluation gaps existed among both country and multilateral organisation PRs [54]. The bulk of the subsequent Global Fund reforms have sought to address financial monitoring shortfalls and to regain the confidence of donors. This has also been supported through additional financial or technical support aimed at widening participation in the financial and programme subfields, enhancing country and community-based monitoring systems, diversifying the range of evaluators, and opening up additional channels for complaints and verification of results. Such support has come from the French government-backed Initiative 5%, GIZ BACKUP Initiative, Grant Management Solutions, and the UNAIDS Technical Support Facility among others.

### The programme subfield

The Fund distinguishes between accountability in finance and programmes, though it recognises that clear links exist between them. The Framework document specifies that “monitoring of Global Fund grants will focus on programmatic accountability: assessing the programmatic progress and public health impact of activities supported by the Global Fund; and providing incentives for improved performance”. CCMs are a critical link between the programme and governance fields. As the Framework Document states [48], CCMs are the “focus for programme accountability, depending on the Board’s decisions regarding overall Global Fund accountability and fiduciary issues”. CCMs coordinate the development and submission of national proposals, nominate the PR and submit requests for continued funding, among other responsibilities.

Once submitted, proposals are reviewed by the TRP, which plays a particularly powerful role in influencing what can be funded. Participation on the TRP is determined by the Strategy, Investment and Impact Committee, itself comprised of representatives from the different donor and implementer blocs on the Board. Given its influential role, it is perhaps not surprising that various criticisms have been directed against its members. There have for instance been accusations that the expert panel members were more sympathetic to donor concerns and that this resulted in a ‘Western’ bias in approving applications from donor-preferred countries, though with little empirical evidence to support such claims [29].

The TRP plays a crucial shaping role in determining what health interventions should be prioritised, and reflects the changing policy priorities of different public and private Board members. This has included approving AIDS treatment interventions at a time when there was resistance from some donors and uncertainty around their eligibility, as well recommending a narrow scope of eligible health systems strengthening activities, scaling back to the disease-specific focus which some viewed as the organisation’s core mandate, though which others criticised [56]. The TRP is not alone in influencing what can be financed, as donors too have used effective veto powers such as withholding funds to bring about programme priorities they favoured. For instance, under the Bush Administration, the US insisted that generic HIV/AIDS medicines had to be cleared by the US Food and Drug Administration before it was willing to pay for them, a move that was seen by some on the Board as delaying decision-making and weakening the participative and deliberative processes of the organisation [57].

Once applications have been approved, grant recipients are monitored as part of a performance-based financing model, an incentive-based mechanism that aims to establish a new standard of accountability [43,48]. As a technical tool, performance-based financing ties future funding to measurement and quantification of results, with countries expected to define the targets and recipients expected to reach them. This model has been viewed as a way for strengthening country management and information systems, as well as creating incentives to scale up programmes and delivery of services [58]. It has simultaneously raised concerns regarding how pressure to meet numerical coverage targets may negatively impact the quality of health services [29]. Indeed, a review of the Global Fund in 2011 recommended that it shift its attention to quality and outcomes rather than focusing on quantity and outputs, and that monitoring and evaluation systems should themselves be reviewed due to data-quality concerns [54].

As noted above, LFAs are contracted by the Global Fund to monitor implementation, verify programmatic results reported by PRs, and to oversee and report on grant performance. As such, they have considerable influence on the programme and decision-making processes of the Global Fund, especially with respect to continuation of funding based on what LFAs report [29]. LFAs have been criticised for relying on results reported by PRs rather than conducting on-site verifications, with the quality of reporting and LFA recommendations coming under serious questioning [59]. Despite their critical role, the work done by many LFAs has been expensive, has not adequately linked fiduciary and programme information, and has often been poorly tailored to the country contexts and specific risks associated with grants [54]. Overall, assessments of LFA performance has shown it to been uneven and inconsistent, with sanctioning mechanisms for firing LFAs for incompetence only in place since 2009 [54,59]. The terms of reference for engaging and approving LFAs were updated in 2013 as part of the Global Fund reforms. In order to be approved by the Global Fund, LFAs are now required to have or be able to access programmatic expertise so as to monitor and link financial and programme performance.

The success of Global Fund programmes is dependent on different actors across all levels, from community to global. At the community level, the organisation rolled out targeted funding for community systems strengthening in 2008 in an effort to increase programme impact through support for community-based organisations and increase participation of these organisations in national programme reviews and evaluations [60,61]. The Global Fund is also dependent on technical and development partners like the WHO, UN Development Programme and other public and private partners to increase programme impact and to support the work of grant recipients. Managing this has sometimes proven difficult. These technical partners are powerful actors in their own right, with their own mandates, budget constraints, accountability structures, sectoral and competing interests. It has often proven politically and institutionally challenging to move beyond the current goodwill-based model of partnership towards more formal arrangements.

In December 2010, the Comprehensive Reform Working Group was established by the Global Fund Board to develop a reform agenda for the organisation. Presenting its report to the Board in May 2011, the Working Group recommended that the Board improve oversight of partnership objectives, review the approach to funding technical assistance, and highlighted the need for formalising agreements and the accountability of partners to the Global Fund and in-country actors [62]. The High Level Independent Review Panel reiterated this need, highlighting that the Global Fund suffers from “inadequate information-sharing and technical cooperation, both at the working level in-country, and inter-institutionally” [54]. Recognizing this, the Global Fund Strategy 2012–16 included reference to the need for improving the provision of technical assistance for programme impact and clarifying relations of accountability at global, regional and country levels as a going concern [47]. The Global Fund has begun to develop or enter into a series of agreements with these partners, including a cooperation agreement with the WHO in May 2014 that formalizes how both organisations will support countries with grant applications in the New Funding Model process.

While the discussion has been primarily focused on accountability relations in the Global Fund programme subfield, a point worth making is that donors, multilateral organisations and initiatives like the Global Fund each exert considerable and competing influence over programme priority-setting and implementation in countries - they can realign the health priorities of countries to reflect their own objectives; yet there is little by way of accountability for the effects of these interventions, regardless of whether they are positive or negative [63]. The monitoring and accountability of these more powerful actors receives significantly less priority than that of less powerful actors, and effective redress mechanisms for holding these and other influential actors to account are limited or lacking at both global and national levels [27,64]. While efforts have been made to advance donor accountability to country-led structures and programmes through mutual accountability compacts, it remains patchy, with donor priorities and activities continuing to be poorly aligned with many country health priorities and processes [17].

### The governance subfield

In terms of governance, the Global Fund has sought to develop a different structure to many other organisations, particularly regarding who participates in its governance activities. The governance of the Global Fund works at two inter-related levels, globally through the Board, and at country level through the CCMs. At the global level, this involves Board membership for a wide spectrum of actors from the public and private sector, some of whom have large delegations to support the work of their representatives, while others do not. The organisation is particularly noteworthy for having given voting rights to NGO delegations and for opening up opportunities for deliberative-decision making. Initially without a vote, the delegation of NGOs representing communities affected by the three diseases gained voting rights on the Board by 2004, giving them the same rights as the two other NGO delegations, states, private sector, and private foundations.

Assessing the robustness of the deliberative processes is difficult, in part due to the complex decision-making and governance arrangements in the Global Fund. Nonetheless, claims have been made suggesting that small groups of powerful states have used formal and informal channels to gain greater advantage in decision-making and priority setting in ways that challenge the deliberative intent of the multisectoral Board [57]. Moreover, concerns have been expressed that the reforms triggered by donors suspending funds in 2011 in the wake of fraud claims have risked alienating civil society engagement from these reform processes, which have instead concentrated on bringing donor confidence back to the organisation [65]. More recent proposals for introducing a tiered pricing framework have received similarly critical reactions, not least because the proposal had apparently been developed in a closed fashion that excluded input from many low and middle-income governments and civil society organisations and where no official information was publicly disclosed [66].

At country level, the Global Fund has tasked CCMs with being the primary mechanism with responsibility for in-country governance, decision-making and coordination. Ideally, CCMs should include a broad representation of state and non-state actors. To this end, the Fund promotes wider participation than many other initiatives at country level, and has exposed participatory weaknesses in existing structures like National AIDS Councils [67]. In opting for having no country presence or office, the Global Fund delegates responsibility to country mechanisms in an effort to promote country ownership for setting priorities and implementing policy, albeit within the confines of the priorities established at the global level of the organisation. The legitimacy of CCMs is therefore dependent on their inclusivity, as well as their performance. Until recently however, CCMs had no formal and regular monitoring arrangement within the Global Fund structure, while engagement with the Secretariat during funding rounds provided only minimal opportunity for assessment of CCM governance activities.

While CCMs have the potential to increase country-ownership and participatory decision-making, in-depth studies have found numerous problems. For example, the report from the High Level Independent Review Panel [54] noted that “all too often, CCMs only pay lip service to inclusive decision-making, and do not exercise genuine or meaningful oversight of grants in action”. In keeping with the light-touch approach of the Global Fund, issues of governance, participation, transparency, selection of financial recipients, efficient resource use and evaluation were often left to the CCMs to establish themselves, which few CCMs did or had the capacity to do [54]. For example, no effective accountability mechanisms or structural safeguards to ensure multisectoral participation were found to have operated within the Peru CCM during the 2004–2007 period [68]. Indeed, no policies were reported to have existed with respect to preventing, defining or managing conflicts of interest even where many representatives on the CCM also represented organisations in receipt of Global Fund finances. Additionally, there was little evidence of effective communication between CCM representatives and those they claimed to represent, resulting in a failure to disseminate information necessary for holding CCM decision-makers to account.

CCMs in several countries have long suffered from participatory and transparency limitations, raising concerns about how participants are selected, how decisions are taken, and whether CCMs undermine or strengthen the legitimacy of the funding process [69,70]. CCMs do at least provide a potential means of formalising greater social participation in proposal development, decision-making and implementation processes. This has in some cases facilitated greater state accountability with respect to increasing access to health services for a wider set of people, as was more recently reported in Peru [71].

While CCMs are cited as a reflection of the Global Fund Board, they also reflect to a great extent the institutional arrangements in countries and relations between actors. Programme weaknesses, governance challenges or wider inequalities that already exist within different country contexts inevitably play out in the activities of the CCMs, including limits on the influence of the community sector [72]. The Global Fund does however have power to tackle some governance challenges in countries, at least where it relates to its own work. Gomez and Atun [73], for instance, argue that the Global Fund played a significant role in transforming health governance in Brazil. Global Fund finances and conditions were found to have raised the profile of the three diseases in ways that increased political commitment to TB in particular, a relatively neglected disease in Brazil. Moreover, it facilitated harmonization of intra-bureaucratic relations, as well as strengthening ties between state and municipal authorities, NGOs and national programmes. Fundamental for increasing participation, Global Fund financing was seen as encouraging “the emergence of new civic movements, participation, and the creation of new municipal participatory institutions designed to monitor the disbursement of funds for Global Fund grants” [73]. The authors caution however that pre-existing political commitment and an effective health movement in Brazil were major factors for governance success, highlighting how context matters and needs to be understood to ensure desired health policy outcomes are realised.

Under the New Funding Model of the Global Fund, minimum standards for core CCM functions will apply and be monitored from January 2015, providing potential opportunities for improving the participation and policy effectiveness of CCMs. Additionally, the Global Fund recently launched a 10 country pilot project to increase engagement of key affected populations in country dialogue processes and in developing concept notes for applying for funding through the New Funding Model. As noted by Garmaise, the “intent of the initiative is to strengthen the ability of CCMs to identify programmatic gaps and intervention needs, and to create “safe spaces” for key affected populations, especially those who are criminalised and marginalised, to engage in the process.” [74]. Enhancing community participation in governance activities may also help to capitalize on the unique opportunity that CCMs offer, namely the potential for gaining a greater degree of input from among a wider set of stakeholders regarding what Global Fund money should be spent on, at least in the context of its disease-specific focus [70,75].

## Conclusion

Accountability is a frequently invoked though arguably less frequently questioned concept in global health cooperation. By critically engaging with the concept and questioning how it is put into practice in light of recent changes to the global health landscape, this paper charts some of the changing dynamics and emerging features of a new accountability agenda. While accountability is about holding actors responsible for their actions, the processes through which this might be done vary substantially, and differ by the policy action area or subfield in which it takes place, whether finance, programme or governance. We suggest that these subfields form the core constituent parts of broader health policy processes and organisational activities; and that each has their own hierarchy of actors and set of policy issues, accountability dynamics and concerns to contend with. It is useful therefore to examine how accountability is practiced in these policy subfields, where different types of accountability battle for recognition and where clear tensions exist. Our focus in this paper is not so much about who ought to be accountable to whom, an important normative line of questioning in its own right. Instead, we have emphasised how accountability in global health is a complex problem, not necessarily reducible to one set of actors holding another to account, but is rather an ongoing process with built-in tensions that continually shape relations between a diverse set of actors.

The Global Fund is representative of the changing dynamics of global health cooperation, and provides a useful case study for illustrating accountability relations in global health policy processes. The organisation is both an accountability seeker and giver, demanding results from those in receipt of its finances, and dependent on the generation and communication of results to ensure financing from its donors. As such, the organisation must manage and is part of a complex system of relations, one in which an inherent double-accountability tension can be observed. The Global Fund’s multi-stakeholder partnership model is challenged by a competing delegation model, that is, a model of accountability where organisational and institutional structures prioritise accountability to those donors who have delegated and entrusted it with powers [29].

This should not however be taken to suggest that donors act as a coherent bloc directing the Global Fund. Indeed, donor constituency members have differed substantially or even clashed behind the scenes regarding the health interventions to be prioritised, and the delivery modalities to be used [20,28,76]. Moreover, donor agencies are influenced in their domestic settings by NGOs, companies and other actors that provide information or lobby intensely for particular policy issues and reforms to be prioritised in foreign aid activities. Within the Global Fund, participation and transparency mechanisms have formalised access for a greater number of actors to influence activities across the financial, programme and governance subfields, not only as donor, NGO or other constituencies, but also through cross-constituency coalitions allied through shared concerns. In a relatively short time, the Global Fund has made a major impact on the three diseases – AIDS, TB and malaria. Its multisectoral partnership model – incorporating stakeholders who previously were not represented at the ‘high table’ – has been credited with facilitating this, which has in turn provided a source of legitimacy for the organisation. Nonetheless, managing a complex system of relations means not only working to regain the confidence of donors by improving accountability in the finance and programme subfields, but also ensuring a strong multisectoral partnership that does not alienate or leave some constituencies missing-in-action from key governance processes.

It is too early to assess if or how the Global Fund has transformed accountability in global health cooperation. However, organisations like the Global Fund who invoke participatory and transparency principles must manage, with some finesse, a complex set of competing accountability relations and mechanisms across different health policy subfields. This is necessary not only for their future financial viability but also for future legitimacy as multisectoral partnerships, particularly if seeking to fulfil participatory promises rather than limiting and instrumentalising the participation of some actors for its own legitimacy purposes.

## Abbreviations

CCMs: Country Coordinating Mechanisms
GHIs: Global Health Initiatives
Global Fund: Global Fund to Fight AIDS, Tuberculosis and Malaria
LFAs: Local Fund Agents
NGOs: Non-governmental organisations
OIG: Office of the Inspector General
PRs: Principal Recipients
TRP: Technical Review Panel
WHO: World Health Organization
